# The Neuro-Ophthalmologic Manifestations of *SPG7*-Associated Disease

**DOI:** 10.3390/jpm15100495

**Published:** 2025-10-16

**Authors:** Ruben Jauregui, Christian Diaz Curbelo, Steven L. Galetta, Scott N. Grossman

**Affiliations:** 1 Department of Neurology, NYU Grossman School of Medicine, New York, NY 10017, USA; christian.diazcurbelo@nyulangone.org (C.D.C.); steven.galetta@nyulangone.org (S.L.G.); scott.grossman@nyulangone.org (S.N.G.); 2 Department of Ophthalmology, NYU Grossman School of Medicine, New York, NY 10017, USA

**Keywords:** optic atrophy, neuro-ophthalmology, SPG7, hereditary spastic paraplegia

## Abstract

The gene *SPG7* codes for the protein paraplegin, a subunit of the m-AAA protease in the inner mitochondrial membrane involved in protein quality control. *SPG7* was initially identified as causing autosomal recessive hereditary spastic paraplegia (HSP), with a pure (insidiously progressive bilateral leg weakness and spasticity) and complex (with additional neurologic features including cerebellar signs and optic atrophy) forms. Now identified as one of the most common causes of HSP, *SPG7*-associated disease has been linked to additional neuro-ophthalmologic features, including isolated dominant optic atrophy, cerebellar eye signs (various forms of nystagmus, dysmetric saccades), progressive external ophthalmoplegia (PEO), and supranuclear vertical palsy. This review describes in detail the various neuro-ophthalmologic presentations of *SPG7*-associated disease, illustrating the role of mitochondrial dysfunction in the pathophysiology of these different entities. Knowledge of the different manifestations of *SPG7*-associated disease is crucial for both neurologists and ophthalmologists, and *SPG7* should be considered in the work-up of patients presenting with entities such as optic atrophy, PEO, and cerebellar eye signs.

## 1. Introduction

Spastic Paraplegia 7 (*SPG7*) is a nuclear gene encoding the protein paraplegin, a subunit of the m-AAA (mitochondrial ATPases Associated with diverse cellular Activities) protease—an ATP-dependent complex in the inner mitochondrial membrane that controls protein quality and regulates ribosome assembly [1]. As a component of the m-AAA protease complex, paraplegin associates with AFG3L2 subunits encoded by the *AFG3L2* gene—mutations in which are responsible for autosomal dominant spinocerebellar ataxia type 28 (SCA28) [1,2]. High levels of paraplegin and AFG3L2 protein expression have been localized to cerebellar Purkinje cells, deep cerebellar nuclei cells, and brain stem motor neurons [3]. It has been demonstrated that loss of paraplegin does not result in the loss of m-AAA protease activity in brain mitochondria, but rather the formation of homo-oligomeric AFG3L2 complexes that are theorized to have dysregulated protease function [1]. Furthermore, loss of the AFG3L2–paraplegin complex impairs the activity of mitochondrial complex I, resulting in increased sensitivity to oxidative stress and elevated production of reactive oxygen species (ROS) [4].

In 1998, De Michele et al. identified pathogenic variants at chromosome 16q24.3, the location of *SPG7*, as the etiology of an autosomal recessive form of hereditary spastic paraplegia (HSP) [5]. Optic atrophy can be associated with HSP, but later studies also identified *SPG7* as the cause of isolated dominant optic atrophy (DOA) and other ocular motor abnormalities, including various forms of nystagmus and ocular duction range limitations (Table 1) [6,7,8]. In this review, we will describe in detail the neuro-ophthalmologic presentations caused by *SPG7*-associated disease.

## 2. Hereditary Spastic Paraplegias: Clinical and Diagnostic Overview

The HSPs are a phenotypically and genetically heterogenous neurodegenerative disorders [5,9]. HSPs have been linked to pathogenic variants across at least 73 genes, with inheritance patterns ranging from autosomal recessive to autosomal dominant and X-linked recessive [10]. Age of onset is mostly in adulthood, though presentations as early as teenage years or as late as the eighth decade have been reported, with the degree of progression varying across genotypes [5,9]. Traditionally, HSPs have been categorized by both the causative gene and the clinical presentation: genes are denoted as “SPG,” followed by a number corresponding to the order in which the entity was discovered [11]. The clinical presentation may be classified as pure or complex [5,12]. Pure HSP cases classically present with insidiously progressive bilateral leg weakness and spasticity, whereas the disease is classified as “complex” when additional features are present, such as cerebellar atrophy/signs, peripheral neuropathy, or neuro-ophthalmologic findings including optic neuropathy, nystagmus, ophthalmoplegia, and ptosis [5,7,12,13].

Given the broad spectrum of clinical presentations, clinicians should maintain a high index of suspicion for HSP in patients presenting with progressive bilateral leg weakness, spasticity, and additional neuro-ophthalmologic features. Additional diagnostic work-up should include MRI imaging with a focus on the posterior fossa, since cerebellar atrophy is frequently observed in patients with complex HSP [14]. A skeletal muscle biopsy can provide the key diagnostic features of ragged red fibers and a mosaic distribution of COX-deficient muscle fibers, suggestive of mitochondrial dysfunction, though these are not seen in all patients [8,15]. In suspect individuals, it is recommended to perform genetic testing, either a multigene panel that includes *SPG7* or more comprehensive testing such as whole-exome sequencing [11,16]. Alternative diagnoses to consider include metabolic disorders, motor neuron disorders, spinocerebellar ataxias, movement disorders, demyelinating disorders, infectious, structural and vascular causes, paraneoplastic syndromes, and nutritional disorders [17].

## 3. *SPG7*-Associated HSP

*SGP7*-associated HSP is most commonly inherited in an autosomal recessive manner, though rare cases with autosomal dominant inheritance have been documented [9,18]. Further studies have also reported that *SPG7* is a common cause of HSP, estimated to account for 4.8% to 13% of HSP patients [19,20]. *SGP7*-associated HSP can present with a pure or complex phenotype, with *SPG7* being the most common etiology in HSP patients with ataxia, found in 9.7% of cases [19].

Important genotype-phenotype correlations have been reported in *SGP7*-associated HSP. In a study of 241 patients, homozygous loss-of-function (LOF) variants presented more often with spasticity/pyramidal signs, optic atrophy, and sensory loss compared to patients with at least one missense variant [21]. In addition, patients with at least one p.Ala510Val variant showed a later onset and more frequent cerebellar ataxia [21]. A different study of 42 patients with biallelic *SPG7* pathogenic variants reported that MRI T2 hyperintensities in the dentate nucleus of the cerebellum, as well as a characteristic phenotype of spastic ataxia were a predictor of *SPG7*-associated HSP [22]. Furthermore, homozygous pathogenic variants in the M41 peptidase domain and c.1529C>T compound heterozygous variants have a younger disease onset [22].

## 4. Neuro-Ophthalmologic Findings

### 4.1. Afferent Visual System

Optic atrophy is the most common afferent neuro-ophthalmologic finding in *SPG7*-associated disease. In the initial study identifying *SPG7* as a causative gene for autosomal recessive HSP, De Michele et al. reported two patients with “pale optic disks,” suggestive of optic atrophy, though without further clinical or paraclinical characterization [5]. Concurrently, Casari et al. included three patients with optic atrophy in their cohort focusing on oxidative phosphorylation deficits in *SGP7*-associated HSP [12]. These two initial studies began to characterize *SGP7*-associated HSP with optic neuropathy as a defining feature [5,12].

It was initially hypothesized that in complex forms of HSP, motor symptoms typically emerged earlier than associated features like optic neuropathy. By contrast, a report by Marcotulli et al. demonstrated that associated features like optic neuropathy can occur before motor symptoms [23]. The report described a patient who was initially diagnosed with congenital optic atrophy, but after developing gait difficulties at the age of 30, he underwent genetic testing as part of a neurologic examination [23]. After excluding variants in mitochondrial DNA and other nuclear genes associated with optic atrophy (e.g., *OPA1*, *AFG3L2*), the compound heterozygous *SPG7* variants p.Val180Met and p.Gly349Ser were identified [23]. The reported examination, including bilateral decreased acuities, pale optic discs, and thinning of the macula ganglion cell-inner plexiform layer (GC-IPL) and peripapillary retinal nerve fiber layer (pRNFL) on optical coherence tomography (OCT), was consistent with optic atrophy [23]. In a similar case, a 57-year-old patient presented with progressive visual decline attributed to a diagnosis of childhood optic nerve atrophy. Examination revealed symmetric light-perception vision, subtle signs of ataxia, and lower extremity spasticity. Genetic testing revealed the homozygous variant p.Met? in *SPG7*, and a diagnosis of complex HSP from *SPG7* was established [24]. A larger study by Klebe et al. exploring the genetic and clinical spectrum in patients with *SPG7* variants reported 10 patients with optic neuropathy, all with thinning of the peripapillary RNFL, variable acuities and disc pallor, with a mean onset of disease in the mid-thirties [7].

The gene *SPG7* has also been shown to cause isolated dominant optic atrophy (DOA). The Klebe et al. study also described a four-generation family with isolated DOA due to the heterozygous *SPG7* variant p.Asp411Ala. The presenting sign of the affected members was decreased visual acuity since the first decade of life, yet they did not report difficulty with walking or leg stiffness, nor did they have the classical findings of HSP [7]. Similarly, a study screened 600 cases of DOA where prior genetic testing for known causes of optic atrophy (*WFS1*, *OPA1*, *OPA3*, and mitochondrial DNA for the three most common LHON variants) was negative, and a total of seven cases exhibited *SPG7* mutations without any of the typical neurologic signs of HSP [25]. Bell et al. also reported a case series of five patients with heterozygous *SPG7* pathogenic variants (three with p.Ala510Val, one with p.Gln447Ter, and one with p.Ala759Thr), all with isolated optic atrophy but without any of the classical symptoms seen with HSP [26]. Overall, these studies demonstrate that heterozygous variants in *SPG7* can cause DOA and provide evidence to support that genetic testing for the work-up of DOA should include the *SPG7* gene.

Clinically, the presentation of *SPG7*-associated DOA resembles other etiologies of optic atrophy, such as DOA from *OPA1* or optic atrophy from Wolfram syndrome [27,28]. Furthermore, most cases present with mild-to-moderate vision loss, though rare reports of patients with poor vision, including hand-motion and count-fingers acuities, exist [6,7,24,25,26]. As expected from optic atrophy, disc pallor and thinning of the RNFL are seen on OCT.

The pathophysiology behind optic atrophy seen in *SPG7*-associated disorders stems from mitochondrial dysfunction. As described above, the protein products of *SPG7* and *AFG3L2* assemble into the mitochondrial m-AAA protease, which plays a critical role in mitochondrial protein quality control [1]. This complex, in turn, regulates the GTPase OPA1, a key mediator of inner mitochondrial membrane fusion and cristae structure integrity [29,30]. Mutations in *OPA1* are known to cause Kjer’s dominant optic atrophy, the most common hereditary optic neuropathy [28,31]. Indeed, optic atrophy is commonly associated with mitochondrial disorders, as the unmyelinated retinal ganglion cells before the lamina cribosa are sensitive to mitochondrial dysfunction due to their high-energy dependence and vulnerability [32]. Optic nerve involvement is also seen in other mitochondrial neurodegenerative conditions, such as Friedreich’s ataxia (*FXN*) or Charcot-Marie-Tooth disease type 2A (*MFN2*) [33,34].

### 4.2. Efferent Visual System

Cerebellar involvement is commonly seen in *SPG7*-associated disease. Cerebellar signs or atrophy on brain imaging are the most frequently observed additional features in complex *SPG7*-HSP patients, while *SPG7* variants have been reported to be a common cause of undiagnosed ataxia [14,35]. Given the integral role that the cerebellum plays in ocular motor control, cerebellar involvement in *SGP7*-associated disease leads to a variety of efferent neuro-ophthalmologic findings [36,37].

Bogdanova-Mihaylova et al. characterized the neuro-ophthalmologic manifestations in a cohort of 32 patients with *SPG7*-associated spastic ataxia [6]. Among the 29 individuals who underwent brain MRI, varying degrees of cerebellar atrophy were observed in all imaged cases. Efferent neuro-ophthalmologic manifestations included gaze-evoked nystagmus (GEN), saccadic pursuits, and dysmetric saccades (hypo- and hypermetric were observed), all considered “cerebellar eye signs” due to their origin from cerebellar pathology [6,36]. Out of 32 patients, 15 presented with GEN, a finding also present in the case of optic neuropathy, spastic paraparesis, and mild cerebellar atrophy reported by Marcotulli et al. [6,23]. Saccadic pursuits, observed in 10 out of the 32 patients, localize to the flocculus/paraflocculus complex of the cerebellum, with possible contribution from the cerebellar vermis [36]. Saccadic dysmetria was identified in 6 patients [36].

In addition to GEN, other forms of nystagmus have also been reported. In the previously described patient by Eriksen et al., who presented with childhood optic atrophy and was found to have the homozygous p.(Met?) *SPG7* variant, pendular nystagmus was evident [24]. Acquired pendular nystagmus is classically seen with multiple sclerosis or with oculopalatal tremor, and is thought to arise from brainstem or cerebellar lesions [38]. Furthermore, Hickman et al. described a patient with leg weakness, spasticity, ataxia, and cerebellar atrophy on imaging, who was also found to have alternating hypertropia, hypermetric saccades, and periodic alternating nystagmus (PAN) that reversed every 130 s [39]. Genetic testing found two compound heterozygous *SPG7* variants, p.Ser576Trp and p.Glu484fs [39]. PAN typically localizes to the cerebellar nodulus/uvula and has been reported in other conditions like ataxia-telangiectasia [36,40].

Other ocular motor abnormalities that are not cerebellar in origin have also been reported. Progressive external ophthalmoplegia (PEO) is a myopathy causing progressive ophthalmoplegia, ptosis, or most frequently both [41]. A study by Pfeffer et al. performed genetic testing on 68 patients with PEO of unclear etiology and discovered 9 patients with compound heterozygous and 6 with heterozygous variants in *SPG7*. The clinical phenotype consisted of ophthalmoplegia causing varying motility range deficits, marked ptosis, and spastic or progressive ataxia, with a mean age of symptom development around 40 years in those with compound heterozygous variants and 26 years in those with single heterozygous variants [8]. In the Bogdanova-Mihaylova et al. study of 32 patients with ataxia from *SPG7*-associated disease, 8 patients were found to also have reduced motility range (upgaze and lateral gaze), and 2 patients had PEO with ptosis [6]. PEO is a mitochondrial disease caused by either mutations, rearrangements, or deletions in mitochondrial DNA (mtDNA), or mutations in nuclear genes that participate in the maintenance of mtDNA (e.g., *POLG*, *TWNK*) [8,42]. Evidence of the mitochondrial dysfunction caused by *SPG7* mutations can be observed in skeletal muscle biopsies of such patients, which demonstrate ragged red fibers and cytochrome *c* oxidase (COX)-deficient muscle fibers [8,12].

Supranuclear palsies have also been associated with *SPG7*-associated disease. In a study of a family with three siblings with complex *SPG7*-HSP, Warnecke et al. reported the presence of convergence failure and vertical eye movement paresis (supraduction greater than infraduction) [43]. Additional examination of the motility deficits revealed that they improved by turning the patient’s head while fixing at a target, making use of the vestibulo-ocular reflex, suggesting that the limited vertical range was supranuclear in origin [43]. A study by Milenkovic et al. of five patients with *SPG7*-HSP also reported a vertical supranuclear palsy in two patients [44]. This team employed oculography to measure saccade velocities and reported that all five patients exhibited slower vertical saccades relative to normal controls, maximal in attempted upgaze [44]. Given the overlapping clinical features with progressive supranuclear palsy (PSP), particularly the presence of vertical supranuclear gaze palsy, the authors also assessed saccade velocities in PSP patients [44]. They found that although saccades were slowed in *SPG7*-associated disease, saccade velocities were significantly slower in PSP [44]. Notably, downward saccades were affected more than upward ones in PSP—a distinguishing feature from SPG7-disease—which supports the utility of oculographic assessment in differentiating between these two entities when diagnosis is unclear [44].

The presence of a supranuclear gaze palsy in *SPG7*-related disease is intriguing, as a vertical supranuclear palsy localizes to brainstem structures such as the posterior commissure and dorsal midbrain (Parinaud syndrome), or to nuclei involved in vertical gaze control, including the rostral interstitial nucleus of the medial longitudinal fasciculus (riMLF) or the interstitial nucleus of Cajal (INC), rather than the cerebellum [45]. The pathogenesis of a supranuclear palsy in *SPG7*-associated disease, however, may also involve mitochondrial dysfunction. For example, multiple studies have suggested that mitochondrial impairment plays a critical role in the pathogenesis of PSP [46,47]. The presence of PSP-like tau depositions in the brainstem of a patient with *SPG7* disease further supports a link between these two conditions [48]. As such, mitochondrial dysfunction from *SPG7*-disease may produce similar impairments in the brainstem that could explain a vertical supranuclear palsy, as seen in PSP.

## 5. Other Mitochondria-Associated Nuclear Genes with Neuro-Ophthalmologic Manifestations

As alluded to above, mitochondrial dysfunction can manifest with various neuro-ophthalmologic disorders and result from mutations in either mtDNA or nuclear genes involved in mitochondrial function, such as *SPG7*. The gene *POLG* (DNA Polymerase Gamma) is a nuclear gene involved in mtDNA maintenance by increasing the fidelity of mitochondrial DNA replication [49]. Pathogenic variants in *POLG* are the most frequent cause of PEO from nuclear DNA, inherited in an autosomal dominant or recessive pattern [41,49]. Patients with pathogenic *POLG* variants can also develop extraocular manifestations, classified as PEO-plus phenotypes, such as ataxia, peripheral neuropathy, and sensorineural hearing loss, with a recurrent form being SANDO (sensory ataxic neuropathy, dysarthria, and ophthalmoplegia) [41,50]. Pathogenic variants in the nuclear gene *TWNK* (Twinkle mtDNA helicase), which encodes a helicase involved in mtDNA replication, have also been associated with autosomal dominant PEO [51]. Other nuclear genes with various mitochondrial roles have also been implicated in PEO, including *SLC25A4*, *RNASEH1*, *POLG2*, *TK2*, and *GMPR* [41]. Although rare, systemic or isolated optic neuropathy can also be a manifestation of *POLG*-associated disease [52]. Both *OPA1* and *WFS* also cause optic neuropathy, in the form of Kjer’s dominant OA and Wolfram syndrome, respectively. Wolfram syndrome classically presents with the “DIDMOAD” tetrad (diabetes insipidus, diabetes mellitus, optic atrophy, deafness), though the diagnostic criteria ismet by the presence of OA and early-onset insulin-dependent DM, since only 50% of patients have the complete phenotype [27,53]. The protein wolframin, encoded by *WFS1*, is localized to the endoplasmic reticulum and plays an important role in protein folding, calcium homeostasis, and regulating mitochondria-associated membranes [54]. Friedreich’s ataxia results from pathogenic variants in the nuclear-encoded *FXN* gene, which produces frataxin—a mitochondrial protein crucial for the assembly of iron-sulfur clusters [33]. Pathogenic variants in the nuclear gene *MFN2* lead to Charcot-Marie-Tooth disease type 2A and encode for the protein mitofusin 2, a mitochondrial outer membrane protein involved in mitochondrial fusion [34]. OA has been reported in both of these neurodegenerative mitochondrial disorders [33,34]. Additionally, the nuclear gene *AFG3L2*, which codes for the AFG3L2 protein that forms the m-AAA protease complex along with paraplegin, is known to cause SCA28 and has also been implicated in non-syndromic isolated dominant OA [55,56]. Various other nuclear genes related to mitochondria have also been reported to cause optic atrophy, such as *ACO2* and *FDXR* [31].

## 6. Conclusions

The presentation of *SPG7*-associated disease is heterogeneous and may present clinically to either neurologists or ophthalmologists. Variants in *SPG7* can lead to both autosomal recessive pure HSP, with insidiously progressive bilateral leg weakness and spasticity, and complex HSP, which often presents with additional features of optic atrophy and cerebellar eye signs. Although rare, a few causes of autosomal dominant HSP have also been reported. Furthermore, autosomal dominant optic atrophy without other neurologic findings can also be seen as the only presenting symptom. The cerebellum is often involved in *SPG7*-disease, leading to various ocular motor abnormalities that localize to the cerebellum, including GEN, PAN, pendular nystagmus, and saccadic dysmetria. Due to involvement of skeletal muscle in mitochondrial disease, PEO and ptosis can also be seen due to variants in *SPG7*. As such, genetic testing for *SPG7* remains an important consideration in the work-up of spastic paraplegia, cerebellar signs or ataxia, and other neuro-ophthalmologic manifestations, including optic atrophy, cerebellar eye signs, and PEO.

## Figures and Tables

**Table 1:** Variousneuro-ophthalmologic manifestations of *SPG7*-associated disease, along with their localization and characteristics.

Manifestation	Localization of Pathology	Description/Characteristic
Optic atrophy (OA)	Optic Nerve	Can be seen as part of the complex phenotype of hereditary spastic paraplegia, or as isolated dominant OA
Gaze-evoked nystagmus	Brainstem neural integrators (nucleus prepositus hypoglossi for horizontal and the interstitial nucleus of Cajal for vertical gaze holding); cerebellar flocculus/paraflocculus complex	Nystagmus triggered in an attempt to hold gaze in an eccentric (non-primary position)
Saccadic pursuits	Cerebellar flocculus/paraflocculus complex, possible contribution from the vermis	Eyes attempt to track using saccades instead of smooth pursuits
Dysmetric saccades	Cerebellar vermis	Inability to accurately target the end point of the saccade, either by under (hypometric) or overshooting (hypermetric) the target
Pendular nystagmus	Brainstem, cerebellar flocculus/paraflocculus complex	Smooth, sinusoidal (resembling a pendulum) nystagmus with no distinct fast or slow phases
Periodic alternating nystagmus	Cerebellar nodulus/uvula	Horizontal jerk nystagmus that reverses direction approximately every 90 s
Progressive external ophthalmoplegia	Extraocular muscles	Weakness from extraocular muscles resulting in limited eye movement range in varying directions
Supranuclear palsy	Brainstem	Impaired voluntary gaze, typically improved by reflexes such as the vestibulo-ocular reflex
Slow saccades	Brain stem	Reduced speed of saccadic eye movements, upwards saccades areaffected more than downwards in *SPG7*-associated disease

## Data Availability

No new data were created.
